# Study protocol: Transition from localized low back pain to chronic widespread pain in general practice: Identification of risk factors, preventive factors and key elements for treatment – A cohort study

**DOI:** 10.1186/1471-2474-13-77

**Published:** 2012-05-25

**Authors:** Annika Viniol, Nikita Jegan, Corinna Leonhardt, Konstantin Strauch, Markus Brugger, Jürgen Barth, Erika Baum, Annette Becker

**Affiliations:** 1 Department of General Practice/Family Medicine, University of Marburg, Karl-von-Frisch-Str. 4, 35043, Marburg, Germany; 2 Institute of Medical Informatics, Biometry and Epidemiology, Chair of Genetic Epidemiology, Ludwig-Maximilians-Universität Munich, Ingolstädter Landstraße 1, 85764, Neuherberg, Germany; 3 Institute of Genetic Epidemiology, Helmholtz Zentrum München – German Research Center for Environmental Health, Ingolstädter Landstraße 1, 85764, Neuherberg, Germany; 4 Institute of Social and Preventive Medicine (ISPM), University Bern, Niesenweg 6, 3012, Bern, Switzerland

**Keywords:** Chronic pain, Widespread pain, Low back pain, Primary care, Resources, Resilience, Coping, Adaptation, Self efficacy

## Abstract

**Background:**

Chronic localized pain syndromes, especially chronic low back pain (CLBP), are common reasons for consultation in general practice. In some cases chronic localized pain syndromes can appear in combination with chronic widespread pain (CWP). Numerous studies have shown a strong association between CWP and several physical and psychological factors. These studies are population-based cross-sectional and do not allow for assessing chronology. There are very few prospective studies that explore the predictors for the onset of CWP, where the main focus is identifying risk factors for the CWP incidence. Until now there have been no studies focusing on preventive factors keeping patients from developing CWP.

Our aim is to perform a cross sectional study on the epidemiology of CLBP and CWP in general practice and to look for distinctive features regarding resources like resilience, self-efficacy and coping strategies. A subsequent cohort study is designed to identify the risk and protective factors of pain generalization (development of CWP) in primary care for CLBP patients.

**Methods/Design:**

Fifty-nine general practitioners recruit consecutively, during a 5 month period, all patients who are consulting their family doctor because of chronic low back pain (where the pain is lasted for 3 months). Patients are asked to fill out a questionnaire on pain anamnesis, pain-perception, co-morbidities, therapy course, medication, socio demographic data and psychosomatic symptoms. We assess resilience, coping resources, stress management and self-efficacy as potential protective factors for pain generalization. Furthermore, we raise risk factors for pain generalization like anxiety, depression, trauma and critical life events. During a twelve months follow up period a cohort of CLBP patients without CWP will be screened on a regular basis (3 monthly) for pain generalization (outcome: incident CWP).

**Discussion:**

This cohort study will be the largest study which prospectively analyzes predictors for transition from CLBP to CWP in primary care setting. In contrast to the typically researched risk factors, which increase the probability of pain generalization, this study also focus intensively on protective factors, which decrease the probability of pain generalization.

**Trial registration:**

German Clinical Trial Register DRKS00003123

## Background

Chronic localized pain syndromes, especially chronic low back pain (CLBP), are common reasons for consultation in general practice. CLBP is usually defined as pain, on most days of a three months period, in the back area from underneath the costal margin to the gluteal fold [1]. In some cases chronic localized pain syndromes can appear in combination with chronic widespread pain (CWP). Wolfe et al. defined CWP as pain in the left and right side of the body as well as above and below the waist plus pain in the axial skeleton [2]. A Swedish cross-sectional-study found a CWP prevalence of 28% out of all female CLBP patients in primary health care [3]. It is still unclear whether CWP is a complication of chronic local pain or an independent pain syndrome [4].

The association of CWP and several physical and psychological factors are well-documented. Studies show high prevalence of comorbidities [5] (most common are anxiety and somatization disorders). CWP patients are more often of older age, female, less educated and have decreased health related quality of life [6]. They show restricted functional ability [7] and frequently receive a disability pension [8]. In addition, there is a high social and economic burden of chronic pain, especially CWP [9]. These studies are population-based cross-sectional and cannot determine the temporal relationship between predictors and the development of CWP (generalization of pain). There are few prospective studies that explore predictors for the onset of CWP. Gupta and colleagues performed a population-based prospective study (EPIFUND-study) [10]. They identified the following disorders to be associated with CWP: psychological distress (in this case anxiety, depression, somatization disorders) [10], health related quality of life [11], physical inactivity [12], and sleep disorders [13].

Previous research, including the study of Gupta et al., primarily focused on risk factors (i.e., negative aspects advancing the development of the disease and increasing the probability of illness [14]). Our aim, on the other hand, is to identify protective factors (predictors decreasing the probability of illness [14]). Our research approach has been derived from the salutogenetic concept of Aaron Antonovsky [15]. His model of salutogenesis focuses on factors enhancing health and wellbeing, which protect (healthy) individuals from potentially damaging influences. Resources like resilience, self-efficacy and coping strategies are under consideration for being protective factors for incident chronic pain syndromes. Costa et al. found a strong relationship between pain self-efficacy, pain intensity and disability in patients with a recent onset of chronic low back pain [16]. Another study by Ong et al. illustrated that psychologically resilient individuals rebound from daily pain catastrophizing through experiences of positive emotions [17]. Finally, a study by Tan et al. studied the relationship between coping, beliefs and the adjustment to chronic pain [18].

General practitioners (GP) are often the main coordinators of CLBP and CWP patients’ management. They provide longitudinal continuity of care and play a major role with respect to appropriate care and health service utilisation [19]. Therefore, epidemiological data and knowledge about distinctive features between CLBP patients and CWP patients is needed, especially for primary care setting. GPs need to have predictors to identify CLBP patients at risk of developing CWP. Knowledge about protective factors will help to support GPs in an individualized treatment approach.

### Aim of the study

The aim of our study is to provide prevalence data on CLBP and CWP for the primary care setting. The differences between both pain localizations, with respect to pain characterisation and comorbidities, will be determined. In addition, we search for risk factors and protective factors of pain generalization in primary care CLBP patients.

## Methods/Design

### Design overview

A 12 month cohort study is performed with baseline data collection (T0) and four follow ups (after 3, 6, 9 and 12 months, T1-T4).

This project is part of the research Consortium LOGIN “Localized and Generalized Muskuloskeletal Pain: Psychobiological Mechanisms and Implications for Treatment“ funded by the German Federal Ministry of Education and Research.

### Study population

For a multi centre data collection, general practitioners in the State of Hessen, Germany are approached. Due to logistical reasons, recruitment is conducted in four waves. Participating practices (doctors and assistant personnel) are asked to recruit all eligible patients who consult for CLBP during a 5 months period, including patients consulting in home visits and emergency calls. Detailed inclusion and exclusion criteria are shown in Table 1. Trained clinical monitors visit the practices to insure validity and consecutive recruitment, during two times (at beginning and at half time of recruitment period).

**Table 1:** Inclusion- and exclusion criteria

**Inclusion criteria**	All patients with chronic* low back pain** as a primary or secondary consulting reason in general practice.
	*”chronic” was defined as pain during most days in the last three months.
	**”low back pain” means pain in the back area under the costal arch, but over the bottom fold (with or without pain radiation).
**Exclusion criteria**	- patients under 18 years
	- pregnant women
	- persons with insufficient fluency of speaking German language or dementia (subjective impressed by doctor)

The number of patients, who deny participation is documented along with the reasons (in an anonymized way), and these patients are accounted for our prevalence analyzes.

### Data collection

A pen and paper questionnaire is used for data collection. After written consent is obtained, patients are asked to fill in the first questionnaire. In addition, general practitioners complete a case report form for every participant. CLBP patients, having no CWP at baseline, are included in the cohort study with a 12 months follow up. They receive mailed follow up questionnaires every three months (T1-T4), and if necessary, followed by two telephone reminders. In cases where participants are unreachable, we do ten calling attempts, during different day times before aborting the reminding process. Participants, who do not return questionnaire and do not explicitly refuse future participation, will be sent the next questionnaire as scheduled.

### Baseline measurements

For investigating distinctive features (e.g. pain characteristics) between CLBP patients and CWP patients as well as potential risk and protective factors, the following physical and psychological parameters are collected at baseline (Table 2):

**Table 2:** Overview of measurement instruments and time of assessment

			**CLBP + CWP**	**CWP**	**CLBP**			
**Function**	**Construct**	**Type**	**T0**	**T1**	**T1**	**T2**	**T3**	**T4**
			baseline	3 months	3 months	6 month	9 month	1 year
**Description of sample characteristics**	Socio demographic data	German Pain Questionnaire (module S)	+					
	Pain anamnesis	Body pain drawing model	+	+	+	+	+	+
		German Pain Questionnaire (pain anamnesis)	+					
		Multidimensional Pain Inventory (part 1, social support subscale)	+					
		Multidimensional Pain Inventory (complete)						+
		Graded Chronic Pain (von Korff Index)	+					
	Pain perception	Schmerzempfindungsskala (SES)		+	+			
	Therapy course + medication	German Pain Questionnaire (therapy and medication items)	+			+		+
	Comorbidities	Self Administered Comorbidity Questionnaire (SACQ)	+					
	Psychosomatic symptoms	Symptom Check-List-90-R (SCL-90-R, Somatisation subscale)	+					
**Risk factors**	Screening: anxiety + depression	Hospital Anxiety and Depression Scale (HADS)	+					
	Trauma Screening	Post Traumatic Diagnostic Scale (PDS-d-1) (part 1 + 2)		+				+
	Critical life events	Questionnaire for critical life events		+				+
**Protective factors**	Resilience	Brief Resilience scale (RS-11)	+					
	Coping resources for back pain	Fragebogen zu Bewältigungsressourcen bei Rückenschmerzen (FBR)	+					
	Stress Management	Brief-Cope		+				+
	Self-Efficacy	GSE (General Self-Efficacy)		+				+

#### Description of sample characteristics - pain characteristics

For pain localisations and consequently for definition of CLBP and CWP we chose the **body pain drawing model** from Pfau et al. [20], where we modified the picture of the head and food. For definition of CWP, the ACR criteria from Wolfe et al. are used [2]. To allow for a standardized, objective analysis and classification we developed an evaluation template according to Diesner et al. [21] and Harkness [22].

We used the **“German Pain Questionnaire”** to determine the characteristics and history of the pain [23]. It is the official pain questionnaire from the **German Association for the Study of Pain**. The questions cover the following aspects: socio demographic data, subjective pain description and perception, factors of pain alleviation and exacerbation, disturbance as a result of pain, subjective pain model, screening for depression and anxiety disorders, health care utilization, as well as medical and psychological comorbidities. Nagel et al. tested the feasibility and content validity in a sample of 3000 patients [24]. Comparison with external criteria (e.g. medical and psychiatric-psychological diagnoses, physician-determined chronicity of pain) showed good content validity and excellent reliability of patients statements [24]. For this study we selected the following modules: pain duration, -characteristics and –course, socio demographic data, health care utilization and medication.

To assess the partner’s reaction in response to patient’s pain, we use the social support subscale (3 items) of the “**West Haven-Yale Multidimensional Pain Inventory**” [25,26]. It is a 12 scales questionnaire measuring the impact of pain on a patient’s life, the responses of others to the patients’ communications of pain and the extent to which patients participate in common daily activities.

The reliability test of the German version (MPI-D) from Flor et al. showed moderate to high internal consistency of the different subscales (α = 0.63-0.90) [26]. For validation, every subscale was correlated with an external instrument showing moderate to high validity [26].

To classify subjective severity of chronic pain we use the “**Graded Chronic Pain**” questionnaire (GCP) according to von Korff [27]. The self-report measure uses ratings of pain intensity and pain related disability. The author suggests a hierarchical model of pain severity where medium pain intensity is represented in the lower range of pain severity, whereas measurements of intense pain together with pain related disability are higher on the scale [28]. This is a 7 item questionnaire resulting in four hierarchical categories: Grade I, low disability - low pain intensity; Grade II, low disability - high pain intensity; Grade III, high disability - moderately limiting; Grade IV, high disability - severely limiting. Grade III and IV usually imply high pain intensity. According to Klasen et al. [28], the German version of the GCP, the factor 'Disability Score' showed a high internal consistency (α = 0.88), whereas the internal consistency of the factor 'Characteristic Pain Intensity' was moderate (α = 0.68) [28]. There is a moderate to high correlation of the German GCP and its subscales to other instruments on the patient's disability (Funktionsfragebogen Hannover: Spearman’s Rho = −0.34; Pain Disability Index: Spearman’s Rho = 0.56) [28].

#### Description of sample characteristics - comorbidities

Comorbidities are assessed by the “**Self Administered Comorbidity Questionnaire**” (SACQ) [29,30]. Participants are asked to comment on 12 frequent medical conditions (heart disease, high blood pressure, lung disease, diabetes, ulcer or stomach disease, kidney disease, liver disease, anemia or other blood disease, cancer, depression, arthritis, and back pain). For each medical condition patients are asked whether they suffer from this problem, if he/she receives treatment for it, and if the problem causes functional limitations. The test-retest reliability for the SCQ was 0.94 (95% confidence interval: 0.72 to 0.99) as calculated by the intra class correlation coefficient [29]. Regarding validity, the Spearman correlation between the SACQ and the Charlson Index was 0.55 for the instrument [29].

#### Description of sample characteristics - psychosomatic symptoms

To identify psychosomatic symptoms, we use the somatization subscale of the “**Symptom Check-List-90-R”** (SCL-90-R) [31]. It is a commonly used psychological status symptom inventory for mental illness. It is built up of nine dimensions: somatization, obsessive compulsive, interpersonal sensitivity, depression, anxiety, anger/hostility, phobic anxiety, paranoid ideation and psychoticism. The instrument supports the computation of three overall scales, comprising the number of symptoms in general and their mean impact. Items are rated on the 5-point Likert Scale of distress, ranging from “not at all” to “extremely”. Schmitz et al. described a high reliability (internal consistency of somatization subscale α = 0.81) and a moderate concurrent validity compared to the “General Health Questionnaire” (0.52) [32].

#### Risk factor - anxiety and depression

The “**Hospital Anxiety and Depression Scale**” (HADS) [33,34] screens for anxiety disorders and depression. It consists of two 7-item subscales (anxiety and depression). Each item scores on a four-point Likert scale and assesses symptoms in the last week. Psychometric properties of the HADS were assessed in numerous studies. Herrmann et al. reported high reliability of the anxiety and depression subscales (internal consistency anxiety α = 0.80; depression α = 0.81) as well as high validity (correlated with external anxiety scales r = 0.65; depression scales r = 0.70) of the German version of HADS [34].

#### Protective factor - coping resources for back pain

We use the FBR-questionnaire “**Fragebogen zu Bewältigungsressourcen bei Rückenschmerzen”** from Tamcam et al. to assess coping resources for back pain [35]. It was specially developed for identifying helpful coping resources of patients with back pain in primary care setting. While other scales rather ask for the knowledge of pain coping skills and resources, the 12-item-FBR explicitly asks for their perceived helpfulness [35]. Items are rated on an eleven-point Likert scale ranging from “not helpful” to “very helpful”.

#### Protective factor - resilience

**Resilience** as a potential protective factor towards pain generalisation is estimated using the resilience scale RS-11, a shortened and validated German form of the Wagnild & Young questionnaire [36,37]. It measures the capacity to withstand life stressors. We use the 11-item short version of the 25-item original scale. It consists of two subscales (Personal Competence and Acceptance of Self and Life). Reliability testing by Schumacher et al. proved high internal consistence (α = 0.91). In addition, validity of RS-11 was demonstrated by a high correlation with the construct of self-efficacy [37].

### Supplementary baseline measurements for CWP patients

CLBP patients, having CWP at baseline, get supplementary concluding measurement instruments (SES, Brief COPE, GSE, PDS-d-1, critical life events, apart from therapy course, medication, MPI and body pain drawing model), like the cohort group gets during follow ups. Taking into account the length of the questionnaires they are assessed after 3 months. An extensive description of the measurement instruments and our motivation for using it is described in the next chapter.

### Follow up measurements for CLBP patients (cohort group)

To identify incidental CWP (primary outcome), we collected data every three months to screen for pain generalization using the **body pain drawing** model (T0-T4).

In addition the following physical and psychological parameters are collected at follow up (Table 2):

#### Description of sample characteristics - pain perception

We assess pain perception with the “**Schmerzempfindungsskala**” (SES) during the first follow up [38]. The questionnaire consists of 24 items with two global dimensions “affective aspects of pain perception” and “sensory aspects of pain perception”. The items ask for different pain perceptions (for example: “My pain feels hot.”) and patients have to judge their degree of agreement on a four-point Likert scale ranging from “not true” to “absolutely true”. Psychometric tests showed high reliability (internal consistency of the affective subscale/sensory subscale α = 0.92/α = 0.81) and good validity [38].

#### Description of sample characteristics - health care utilization and medication

Just like at baseline, **health care utilization and medication** during the preceding half year are assessed after the 6th and 12th month (T2 and T4).

During the 12 months follow up the following data will be collected:

#### Protective factor - coping

To assess stress management competence (coping), we use the **Brief COPE** from Carver et al. [39,40]). It is a shorter version of the original COPE questionnaire and consists of 14 subscales, where each subscale is represented by two items. Participants are asked to think of their usual thoughts and actions while faced with a difficult situation and to indicate their answers on a four-point Likert-type scale, rating the resemblance of each item to coping efforts pursued. The response scale ranges from “not at all”, “a little bit”, “considerably” to “very much”. Cooper et al. tested reliability of Brief COPE to patients with Alzheimer’s disease. They assessed average internal consistencies for emotion-focused (α = 0.72), problem-focused (α = 0.84), and dysfunctional (α = 0.75) subscales [41]. Regression analyses indicate convergent and concurrent validity [41].

#### Protective factor - self-efficacy

We use the “**general self-efficacy-questionnaire”** (GSE) to assess self-efficacy [42,43]. It applies to a patient’s self-assessment of their ability to improve their daily troubles and how they cope with critical situations. The questionnaire consists of 10 items to be rated by four-point-Likert-scale ranging from “very uncertain”, “rather uncertain”, “rather certain” to “very certain”. Hintze et al. showed a high reliability (internal consistency α = 0.92) and good validity (correlation with the resilience scale r = 0.68) of the German version of GSE [43].

#### Risk factor – trauma

We use Part 1 of the “Posttraumatic Diagnostic Scale” (PTDS) [44] to assess whether patients suffer from traumatic life events. It is a self-report screening instrument based on the type, time duration, and circumstances of traumatic experiences [45]. Griesel et al. reported high reliability (internal consistency of the total scale was α = 0.94) and high validity (correlation with the “Clinician Administered PTSD Scale” (CAPS) r = 0.76; p = 0.001) of the German PTDS [45].

#### Risk factor - critical life events

The **critical life events** questionnaire from the work of Leist et al. are used [46]. Participants are asked to select if and when they experienced any possibly life changing events on a 22 items list.

#### Description of sample characteristics - pain characteristics

Finally, during the fourth follow up measurement point (T4) participants are asked to complete the “**West Haven-Yale Multidimensional Pain Inventory**” [25,26]. This is done to investigate if our CLBP patients can be classified into the three clusters (dysfunctional, interpersonally distressed, adaptive copers) that have been widely shown to represent different subgroups of patients with fibromyalgia [47].

### General practitioners case report

GPs are asked to comment on the individual presence of red flags with every patient included in the study. Red flags are frequently used risk factors for identifying serious disorders causing low back pain [48]. In addition, we ask GPs for their personal opinion about whether the patient’s prognosis will be better, equal or worse in one year. GPs are asked to estimate each patient’s probability for pain generalization during the follow up.

### Qualitative sub-study

Based on the results of the cohort study a qualitative sub-study will be performed, after completed follow up, regarding patients’ and physicians’ perspectives of a resource based treatment approach for prevention and therapy of CLBP and CWP.

### Statistical analysis

Group comparisons of CWP and CLBP patients will be performed. For this we use measures of central tendency (mean and standard deviation, median, percentile, frequency and percentages) as well as chi-square tests for categorical data. The *t*-test is applied for comparing means. Effect sizes will be expressed in terms of odds ratios for categorical dependent variables and, for quantitative dependent variables, in terms of group differences or differences per unit change of a quantitative predictor variable.

For identification of predictors logistic regression analysis will be performed using incidental CWP as dependent variable. We use baseline risk and protective factors as independent variables or predictors. All analyses are supervised and advised by a senior statistician of the Institute of Genetic Epidemiology (IGE), University of Munich and Helmholtz Zentrum München.

Focus groups: The results of the focus groups are analyzed according to predefined main topics. Answers of patients and physicians are contrasted.

### Sample size

Based on results from previous studies we presumed a CWP prevalence of 10% [49] leading to 900 patients to be included in the cohort study (localized chronic pain only). Assuming a 10% rate of patients developing generalized pain (n = 90) [50] up to 9 independent variables may be included in the logistic regression analysis (corresponding to 10 events per independent variable [51,52]).

### Power calculation

Assumptions: Prevalence of generalized chronic back pain of 10%. A change of an independent variable by one standard deviation in the population implies an odds ratio of 1.65. Calculated power: 0.83 for alpha = 0.005, which means after adjustment of multiple testing of 9–10 variables an overall probability of the type one error of 0.05 will not be exceeded. The power has been calculated by performing simulations using the software R (R Development Core Team 2010).

### Ethics committee

The study was approved by the local ethics commission of Philipps-University Marburg (Ethik: 11.06.2010, AZ 88/10) and is in accordance with the Declaration of Helsinki.

## Discussion

This cohort study will be the largest study which prospectively analyzes predictors for transition from CLBP to CWP in primary care setting. In contrast to the typically researched risk factors, which increase the probability of pain generalization, this study also focus intensively on protective factors, which decrease the probability of pain generalization. The identified risk and protective factors might aloud GPs to identify patients at risk for development of CWP. Furthermore, the acquired knowledge about protective factors will help us to develop an individualized treatment approach for GPs.

Selection bias is often the major limitation of a cohort study. The amount of psychological constructs measured in this study can be rather demanding in terms of endurance and intellectual abilities compared to usual studies in primary care setting. For our primary care study population, which includes a high proportion of older people and persons with lower educational background, it may be a problem to deal with the questionnaires. These patients might be more likely to deny participation or abort follow up.

It might be possible, that GPs are more likely to remember study recruitment, in special cases (e.g. patients with higher disease severity or special characters) or during times with a lower workload. An incomplete recruitment brings forth the risk of selection bias and lowers external validity.

Results of the baseline analysis are expected in the course of this year (2012). Follow up data will be completed, next year (2013).

## Abbreviations

CLBP: Chronic low back pain; CWP: Chronic widespread pain; GP: General practitioner; MPI: West Haven-Yale Multidimensional Pain Inventory; MPI-D: German version of the West Haven-Yale Multidimensional Pain Inventory; GCP: Graded Chronic Pain; SACQ: Self Administered Comorbidity Questionnaire; SCL-90-R: Symptom Check-List-90-R; HADS: Hospital Anxiety and Depression Scale; FBR: Fragebogen zu Bewältigungsressourcen bei Rückenschmerzen (questionnaire for assessing coping resources for back pain); RS: Resilience; SES: Pain perception scale; COPE: Measurement instrument for coping; GSE: General self-efficacy-questionnaire; PTDS: Posttraumatic Diagnostic Scale.

## Competing interests

**Annika Viniol** does not state any financial or non-financial conflicts of interest.

**Nikita Jegan** does not state any financial or non-financial conflicts of interest.

**Konstantin Strauch** does not state any financial or non-financial conflicts of interest.

**Markus Brugger** does not state any financial or non-financial conflicts of interest.

**Jürgen Barth** does not state any financial or non-financial conflicts of interest.

**Erika Baum** does not state any financial or non-financial conflicts of interest.

**Corinna Leonhardt** does not state any financial or non-financial conflicts of interest.

**Annette Becker** was a consultant for Grünenthal GmbH, from whom she has also received a speaker’s fee.

## Authors’ contributions

Annika Viniol collected and analyzed data and wrote the manuscript. Nikita Jegan collected and analyzed data. Konstantin Strauch and Markus Brugger gave statistical support and planned the study. Jürgen Barth and Erika Baum planned and revised study design. Corinna Leonhardt and Annette Becker planned the study, discussed the results and revised the manuscript critically. All authors edited the drafted version of the manuscript. All authors read and approved the final manuscript.

## Pre-publication history

The pre-publication history for this paper can be accessed here:

http://www.biomedcentral.com/1471-2474/13/77/prepub
